# A universal real-time assay for the detection of *Lyssaviruses*

**DOI:** 10.1016/j.jviromet.2011.07.002

**Published:** 2011-10

**Authors:** David T.S. Hayman, Ashley C. Banyard, Philip R. Wakeley, Graeme Harkess, Denise Marston, James L.N. Wood, Andrew A. Cunningham, Anthony R. Fooks

**Affiliations:** a Rabies and Wildlife Zoonoses Group, Veterinary Laboratories Agency – Weybridge, Woodham Lane, Surrey KT15 3NB, UK; b Cambridge Infectious Diseases Consortium, Department of Veterinary Medicine, University of Cambridge, Madingley Road, Cambridge CB3 0ES, UK; c Institute of Zoology, Zoological Society of London, Regent's Park, London NW1 4RY, UK; d Department of Biology, Colorado State University, Fort Collins, CO 80523, USA; e The National Centre for Zoonosis Research, University of Liverpool, Leahurst, Chester High Road, Neston CH64 7TE, UK

**Keywords:** *Lyssavirus*, Rabies, Bat, SYBR Green, Real-time PCR, Synthetic DNA

## Abstract

*Rabies virus* (RABV) is enzootic throughout most of the world. It is now widely accepted that RABV had its origins in bats. Ten of the 11 *Lyssavirus* species recognised, including RABV, have been isolated from bats. There is, however, a lack of understanding regarding both the ecology and host reservoirs of *Lyssaviruses*. A real-time PCR assay for the detection of all *Lyssaviruses* using universal primers would be beneficial for *Lyssavirus* surveillance. It was shown that using SYBR^®^ Green, a universal real-time PCR primer pair previously demonstrated to detect European bat *Lyssaviruses* 1 and 2, and RABV, was able to detect reverse transcribed RNA for each of the seven virus species available to us. Target sequences of bat derived virus species unavailable for analysis were synthesized to produce oligonucleotides. *Lagos Bat*-, *Duvenhage*- and *Mokola virus* full nucleoprotein gene clones enabled a limit of 5–50 plasmid copies to be detected. Five copies of each of the synthetic DNA oligonucleotides of *Aravan*-, *Khujand*-, *Irkut*-, *West Caucasian bat*- and *Shimoni bat virus* were detected. The single universal primer pair was therefore able to detect each of the most divergent known *Lyssaviruses* with great sensitivity.

## Highlights

► Universal real-time PCR primer pair demonstrated to hybridize to and detect each of the known *Lyssaviruses* (including *Rabies virus*) with greater sensitivity than a standard pan-*Lyssavirus* hemi-nested RT-PCR typically used. ► Target sequences of bat derived virus species unavailable for analysis (*Aravan*-, *Khujand*-, *Irkut*-, *West Caucasian bat*- and *Shimoni bat virus*) were synthesized to produce oligonucleotides and the synthetic DNA was used as a target for primer hybridization.

## 1 Introduction

*Rabies virus* (RABV), genus *Lyssavirus*, family *Rhabdoviridae*, is enzootic throughout most of the world. The domestic dog (*Canis familiaris*) acts as the principal vector (Knobel et al., 2005, 2007), however, a range of mammalian carnivores also can act as hosts (Childs and Real, 2007; Davis et al., 2007; Hass and Dragoo, 2006; Nel et al., 1993; Real et al., 2005; Rupprecht et al., 1995; Swanepoel et al., 1993; Velasco-Villa et al., 2005; von Teichman et al., 1995). Rabies remains the only disease considered to have a 100% mortality rate and millions of animals are killed each year from both the disease and control programmes (Knobel et al., 2005). It is now widely accepted that RABV had its origins in bats and, with the exception of Mokola virus (MOKV), all known *Lyssaviruses* have been isolated from bats (Badrane and Tordo, 2001; Kuzmin et al., 2003, 2005, 2010).

The *Lyssavirus* genus can be differentiated into 11 genetically divergent species based on genetic analyses of the viral genome (ICTV, 2009; Kuzmin et al., 2005). The species are: *Rabies virus* (RABV; *Lagos bat virus* (LBV); *Mokola virus* (MOKV); *Duvenhage virus* (DUVV); *European bat Lyssavirus-1* (EBLV-1); *European bat Lyssavirus*-2 (EBLV-2), *Australian bat Lyssavirus* (ABLV), *Irkut virus* (IRKV), *Aravan virus* (ARAV), *Khujand virus* (KHUV) and *West Caucasian bat virus* (WCBV) (ICTV, 2009). A twelfth genetically related virus, *Shimoni bat virus* (SHIBV) is yet to be classified, but may become recognised as a new species (Kuzmin et al., 2010). SHIBV shares approximately 80% nucleotide identity with other *Lyssaviruses*. Recent studies have also shown LBV phylogeny to be more complex than first thought and four distinct LBV lineages are now reported with high levels of sequence divergence amongst them (Delmas et al., 2008; Kuzmin et al., 2010; Markotter et al., 2008).

The *Lyssaviruses* can further be grouped into phylogroups according to relative antigenicity and sequence diversity (Badrane et al., 2001). Phylogroup I includes all known species apart from LBV, SHIBV, MOKV and WCBV. Along with DUVV, the phylogroup II viruses, LBV, MOKV and SHIBV, have solely African distributions. WCBV awaits classification with regards to phylogroup, but has been proposed as a new phylogroup (III) (Hanlon et al., 2005; Kuzmin et al., 2005). It is important to note that vaccines derived from RABV strains confer little or no protection against members of phylogroups II and III in experimental studies (Badrane et al., 2001; Brookes et al., 2006; Hanlon et al., 2001, 2005; Weyer et al., 2008).

In order to understand virus diversity, ecology and virus-host relationships in bats and other mammalian orders, molecular tools which are able to detect each of the *Lyssaviruses* are required. In the Americas, for example, only RABV circulates; however elsewhere the situation is less clear. For example EBLV-1 (phylogroup I) and WCBV (putative phylogroup III) have been isolated from (WCBV), or nucleic acids detected in (EBLV-1) bats of the genus *Miniopterus* (Banyard et al., 2011). In addition, a member of this genus has been implicated in DUVV transmission in Africa (Sabeta et al., 2007). This genus of bat occurs throughout much of Africa, as well as Eurasia. Virus isolations for these *Lyssaviruses* have been made on different continents with LBV and DUVV being isolated from bats in Africa, and WCBV from a bat in the Caucasus in Russia. Subsequent studies, however, detected anti-WCBV virus neutralising antibodies (VNAs) in *Miniopterus* bats in Africa, suggesting that this virus infects bats over a broad geographical area (Kuzmin et al., 2008b). Viruses belonging to phylogroups I and II (LBV and DUVV) have also been isolated from African bats of the *Nycteris* genus (King et al., 1994; Kuzmin, 2008; Kuzmin et al., 2005). It is noteworthy that sera containing VNAs are often able to cross-neutralise viruses within the same phylogroup (Brookes et al., 2005; Wright et al., 2010). Both anti-LBV and anti-MOKV VNAs, for example, have been detected in *E. helvum* in which LBV is thought to circulate. Although probably representing cross neutralisation by LBV positive sera, co-infection with MOKV or the discovery of other phylogroup II viruses, such as SHIBV, cannot be ruled out in these populations and broadly sensitive assays are required for surveillance studies (Dzikwi et al., 2010; Kuzmin et al., 2008a, 2010; Wright et al., 2010).

Previously, several real-time reverse transcription RT-qPCR assays for the detection of RABV have been described, including a TaqMan^®^ assay for the detection and discrimination of RABV from EBLV-1 and EBLV-2, and an assay designed to detect, but not discriminate amongst, DUVV, RABV, LBV and MOKV (Coertse et al., 2010; Hoffmann et al., 2010; Wakeley et al., 2005). These assays have been shown to be more sensitive than conventional nested or hemi-nested RT-PCR. These assays, however, require numerous cocktails of primers and TaqMan^®^ probes, or else have only been used to detect specific target species. We therefore wished to design a real-time assay that could detect each of the *Lyssaviruses*, in order to enable researchers to use this assay for surveillance studies where the *Lyssavirus* species present is unknown, or there may be numerous viruses circulating in putative reservoir hosts. We therefore describe the development of a SYBR^®^ Green (Applied Biosystems, Foster City, CA, USA) application using two pre-existing PCR primers (JW12 and N165-146), which have been validated to detect RABV, EBLV-1 and -2 (Wakeley et al., 2005), to develop a rapid and sensitive real time assay for the detection of all *Lyssaviruses*. These primers target the nucleoprotein (N) gene, which is the most abundant transcript generated during infection and which includes areas that remain highly conserved across the species. For those newly described *Lyssaviruses* that were not available for analysis from infected material, viral cDNAs were synthesized to determine if they could be detected using the JW12 and N165-146 primers. We demonstrate that these universal primers are able to target the complementary genome of each of the known *Lyssavirus* species.

## 2 Materials and methods

### 2.1 RNA samples

To compare the sensitivity of different PCR assays, total RNAs were extracted from experimentally infected mouse brain tissues from the Veterinary Laboratories Agency (VLA) archive (Table 1). Extractions were performed using TRIZOL™ (Invitrogen™, Paisley, United Kingdom) and RNA was resuspended in DEPC-treated water to 1 μg/μl. LBV isolates extracted included three suggested LBV lineages (A, B, C) (Markotter et al., 2009).

### 2.2 Reverse transcription

Ten-fold dilution series (1–1 × 10^−8^ μg/μl) of total RNA of each sample were made in nuclease-free water. Total RNA (2 μL) was reverse transcribed using Moloney murine leukemia virus reverse transcriptase (MMLV-RT) and the primer JW12, at 42 °C for 60 min, as published previously (Heaton et al., 1997; Wakeley et al., 2005). The cDNA was diluted 1:10 in nuclease-free water.

### 2.3 Hemi-nested PCR

Complementary DNA samples were analysed using a hemi-nested (hn) PCR incorporating pan-*Lyssavirus* primers (JW6/12, JW10/12) (Heaton et al., 1997) for comparative limit of detection purposes and sequencing. Note that the JW12 primer is identical in both this and the real-time assay.

### 2.4 Sequencing

Hemi-nested PCR products derived from a selection of samples were sequenced to ensure that the cDNA used in both hn- and qPCR were the correct viruses and shared the phylogenetic relationships with those previously described (Markotter et al., 2008). All RT-qPCR tests were performed using the same cDNA as that used for the hnRT-PCR from which the sequence data were derived. Direct consensus DNA sequencing of a 405 bp region of the N gene was undertaken as previously described (Johnson et al., 2003). Sequences produced were edited using SeqMan (DNASTAR Lasergene^®^, Madison, WI, USA) and aligned (ClustalW, Megalign, DNASTAR Lasergene^®^, Madison, WI, USA).

### 2.5 SYBR^®^ Green qPCR

Real-time PCR methods using JW12 and N165-146 primers previously described (Wakeley et al., 2005) were adapted for use using the dsDNA dye SYBR^®^ Green (Applied Biosystems, Foster City, CA, USA). The primers were designed against all the *Lyssavirus* species available at the time of the original study (*n* = 7 species) using N gene sequences from 557 viruses (Wakeley et al., 2005). Primer sequences and locations used in this study are given in Table 2.

**Fig. 1:**
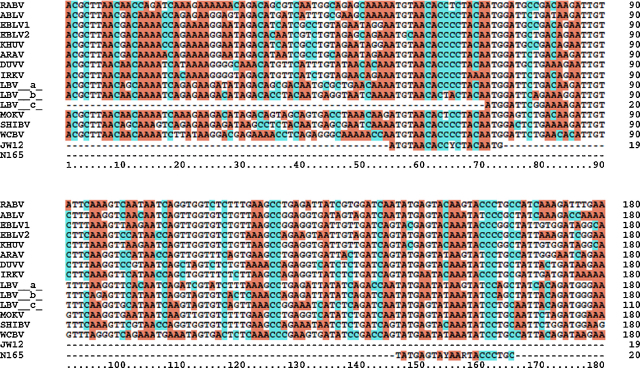
Alignment of all *Lyssavirus* species to date with JW12 forward and N165-146 reverse primer. Dots represent identity to consensus sequence, hyphens are gaps for optimal alignment. Primer region is underlined for clarity. Nucleoprotein start sequence is in bold. Position 1 is the start of the genome. Sequences used in this alignment are as follows: RABV is PV (NC001542), LBV (a) is 0406SEN (EU293108), LBV (b) is 8619NGA (EU293110), LBV (c)* is LBVSA1980 (EF547457), MOKV is 86101RCA (EU293118), DUVV is 94286SA (EU293120), EBLV1 is RV9 (EF614261), EBLV-2 is RV1333 (EF157977), ABLV (AF418014), ARAV (EF614259), IRKV (EF614260), KHUV (EF614261), WCBV (EF614258) and SHIBV (GU170201). *Please note: genome sequence was not available for this sub-species, therefore full N was used instead.

Consensus sequences for the N gene were generated for all *Lyssavirus* species using MegAlign (DNASTAR Lasergene^®^, Madison, WI, USA). The consensus sequences were then compared by using the same software to ensure the universal primers JW12 and N165-146 would anneal to each *Lyssavirus* species (Fig. 1).

SYBR^®^ Green Hot Start (Applied Biosystems, Foster City, CA, USA) PCRs were performed using 20 μL SYBR^®^ Green Hot Start, 13 μL nuclease-free water, 1 μL JW12 (20 pmol/μL) primer, 1 μL N165-146 (20 pmol/μL) primer, and 5 μL cDNA. Amplification was according to the following heating and cooling program: 1 cycle of 94 °C for 2 min followed by 40 cycles of 94 °C for 30 s, 55 °C for 30 s, and 72 °C for 30 s, followed by one cycle each of 95 °C for 1 min, 55 °C for 30 s, and 72 °C for 30 s. The reactions were carried out in Thermo-Fast 96-well PCR plates or Thermo-tube strips with Ultra Clear caps (ABgene^®^, Thermo Scientific, Epsom, United Kingdom) in an MX3000P multiplex quantitative PCR system (Stratagene, La Jolla, CA, USA). For each RT-PCR, a critical threshold cycle number (*CT*) was determined corresponding to the PCR cycle number at which the fluorescence of the reaction exceeded a value determined to be statistically higher than the background by the software associated with the MX3000P system (Stratagene, La Jolla, CA, USA). In addition, melting curve analyses and gel electrophoresis were performed to ensure false positive results, such as those due to primer dimers, were not included in any results. Negative controls were included for each row of samples. A single RABV control (CVS-11, GenBank AB069973) was included in each run with samples of virus isolation origin, but not with those using plasmids.

### 2.6 Sensitivity analysis

Limit of detection analyses were performed from the same cDNA for both the qPCR using JW12 and N165-146 and the previously published hn-PCR.

For those viruses not included in previous studies (Wakeley et al., 2005), real-time PCR sensitivity was also determined by producing full-length N gene blunt ended PCR products and inserting these into a pCR^®^-Blunt II-TOPO^®^ plasmid vector (Invitrogen™, Paisley, United Kingdom) to produce standard controls and templates for *in vitro* transcription of RNA using the MEGAScript kit (Ambion^®^, Foster City, CA, USA). For species which viruses themselves were unavailable, cDNA sequences from GenBank were synthesised (see below).

To produce the N gene clones, total RNA was isolated as described above from cell-cultured isolates of MOKV (RV4), DUVV (RV131) and three suggested LBV lineages (A, RV41; B, RV1; and C, RV3). RNA was reverse transcribed using SuperScript III™ First-Strand Synthesis System for RT-PCR (Invitrogen™, Paisley, United Kingdom) and random hexanucleotide primers. Lineage specific reverse primers and JW12 (Table 2) were used to produce blunt ended PCR products, which were purified and cloned using the Zero Blunt^®^ TOPO^®^ PCR Cloning kit (Invitrogen™, Paisley, United Kingdom). Plasmids were analysed using M13 primer PCR to check for appropriate orientation and that the correct gene termini were present.

Plasmid DNA template was linearised using either *Hinc II* or *KpnI* restriction enzyme (Promega Corporation, Madison, WI, USA). Digests were terminated and *in vitro* transcription performed (MEGAscript^®^, Ambion^®^, Foster City, CA, USA) using T7 or SP6 promotor primers, depending on the inserted nucleoprotein orientation, to synthesize RNA. DNase (MEGAscript Kit^®^, Ambion^®^, Foster City, CA, USA) was added for a 15 min incubation at 37 °C to remove the DNA template. RNA was recovered using Lithium chloride precipitation. Hemi-nested PCRs were performed as above with and without reverse-transcription, to ensure no DNA was contaminating the RNA and DNase treatment was repeated as necessary. The RNA yield was quantified by UV light absorbance (NanoDrop Spectrophotometer, Thermo Scientific, Epsom, United Kingdom) and serial dilutions were made of each lineage RNA and plasmid. The comparative sensitivity of the SYBR^®^ Green (Applied Biosystems, Foster City, CA, USA) was studied using the serial dilutions. For all sensitivity analyses, the DNA or RNA copy numbers detected was deduced from the amount of DNA or RNA added to the PCR, the molecular weight of the nucleic acids and Avogadro's number.

### 2.7 Synthetic DNA

The 40–180 base region of each of the bat derived viruses unavailable but published in GenBank were aligned with JW12 and N165-146 (Fig. 1) and 20 nmole Ultramer DNA oligonucleotides were synthesized (Integrated DNA Technologies, Coralville, IA, USA). A T7 promoter region for RNA transcription and an ATCGATCG leader in the 5′-3′ direction were included to allow efficient primer binding and for future studies. Ultramer DNA was serially diluted 10-fold in RNase free water and SYBR^®^ Green (Applied Biosystems, Foster City, CA, USA) PCR with JW12 and N165-146 primers performed as above.

## 3 Results

Viral nucleic acids from each of the *Lyssaviruses* available were detected by the universal primers using a SYBR^®^ Green (Applied Biosystems, Foster City, CA, USA) qPCR assay when RNA was extracted from infected material (Table 3). The qPCR products produced were detectable by both SYBR^®^ Green (Applied Biosystems, Foster City, CA, USA) and gel electrophoresis (data not shown). Hemi-nested PCR products from RABV, LBV, MOKV, DUVV, EBLV-1, EBLV-2 and ABLV have all previously been successfully reverse transcribed, amplified, and 405 bp regions of the N-gene sequenced using the hnRT-PCR. In this study, this was extended to include viruses from each of the three suggested LBV lineages (A, B and C).

The comparative sensitivity of a qPCR assay using this primer pair has already been assessed for RABV, EBLV-1 and -2. Therefore, in this study the comparative sensitivity of the primer pair was assessed against the hnRT-PCR assay using SYBR^®^ Green using serial dilutions of LBV lineages A, B and C, DUVV and MOKV from archived material. The SYBR^®^ Green (Applied Biosystems, Foster City, CA, USA) PCR assay was typically shown to be approximately 200-fold more sensitive than the previously published hnRT-PCR, depending on *Lyssavirus* species and lineage (Table 3), a similarly greater sensitivity to that for the RABV, EBLV-1 and -2 qPCR assay using the same primers (Wakeley et al., 2005).

LBV, DUVV and MOKV full N gene sequences were successfully cloned into a pCR^®^-Blunt II-TOPO^®^ plasmid vector with matching consensus sequence for the virus used. Using the species specific N gene clones as templates the limit of detection for the SYBR^®^ Green (Applied Biosystems, Foster City, CA, USA) PCR assay was calculated. This was shown to be 5–50 plasmid copies. An approximate 28-fold sensitivity was lost during the RT step (Table 3).

*Lyssaviruses* isolated on single occasions from Eurasian bats and, in SHIBV's case, an African bat, were not available for testing in this study. However, sequence alignment *in silico* suggested that the primer set JW12 and N165-146 would successfully prime off all virus nucleic acids analysed (Kuzmin et al., 2005) (Fig. 1). To test this, DNA was synthesized commercially using published sequences and serially diluted in 10-fold dilutions and DNA was detected to a lower limit of 5 copies for each virus.

## 4 Discussion

The SYBR^®^ Green qPCR assay in this study has been developed to demonstrate that the universal primer pair, JW12 and N165-146 targets the N gene successfully for each of the known *Lyssavirus* species. We developed this to address the problems associated with the diagnosis of divergent *Lyssavirus* infections. There are currently numerous assays available for rabies and *Lyssavirus* detection and these have recently been reviewed (Fooks et al., 2009). Of the several real-time PCR assays published, however, none has been validated against for the detection of all published *Lyssavirus* species. Despite the need for further assessment of the sensitivity of this assay, including as a possible one-step technique, we have demonstrated that this primer pair in a real-time application is more sensitive for the detection of all *Lyssavirus* cDNA that we tested, when compared with a known pan-*Lyssavirus* hnRT-PCR. For some genetically similar viruses, e.g. RV2 and RV611 from LBV lineage C, there appeared to be substantial differences in limits of detection by qPCR. Whilst standardising for total RNA, the true quantity of virus RNA is unknown in these samples. Using plasmid clones addresses this somewhat (see below), however, the substantial differences in detection between the qPCR and hnRT-PCR for LBV lineage C viruses may reflect poor primer binding of the hnRT-PCR for LBV viruses RV2 and RV3, compared to the qPCR primer pair. This has been discussed previously by another group (Coertse et al., 2010). Further evidence of the increased primer binding efficiency for this universal primer pair has been previously demonstrated on a panel of over 40 RABV and a range of EBLV-1 and -2 viruses (Wakeley et al., 2005), which demonstrated a similarly greater sensitivity over the hnRT-PCR to that found in this study. This new assay overcomes restrictions in the current hnRT-PCR assay which requires multiple transfers of material and substantial time (both in required man-hours for preparation and thermal cycling) (Heaton et al., 1997). Despite this, the hnRT-PCR has been sufficient to detect virus from each virus-positive brain sample and therefore still offers a useful tool for rabies diagnosis where conventional PCR technology exists. Importantly, hnRT-PCR produces a PCR product that is readily sequenced.

The use of only two universal primers (JW12 and N165-146) plus SYBR^®^ Green (Applied Biosystems, Foster City, CA, USA) dsDNA dye is an easy to use assay which enabled each of the viruses archived in the WHO and OIE rabies reference laboratory at VLA to be detected in infected brain material. The PCR assay was shown to be highly sensitive, detecting between 5 and 50 copies of N-gene target sequences when cDNA from MOKV, DUVV and LBV (lineage A, B and C) were cloned into plasmid vectors and 25–195 copies of LBV RNA generated by *in vitro* transcription. Sensitivity for MOKV and DUVV RNA was lower, with 190–1430 copies detected. In each case, when used on infected material, the assay was more sensitive than the previously published hnRT-PCR, however future studies should assess the RNA detection limit for each of the viruses known. Additional attempts to further optimise the RT step, which leads to a loss of sensitivity, should also be made.

The use of synthetic DNA for the *Lyssaviruses* isolated on single occasions from bats is novel and demonstrates that this JW12/N165-146 primer pair is able to detect cDNA from all currently known species. The detection limit was good; with the JW12/N165-146 SYBR^®^ Green assay able to detect 5 DNA copies of DNA Ultramers. This JW12/N165-146 primer set has previously been described for use with RABV, EBLV-1 and EBLV-2 TaqMan^®^ probes (Wakeley et al., 2005) and in this study was shown to detect ABLV cDNA when used in conjunction with the dsDNA dye, SYBR^®^ Green (Applied Biosystems, Foster City, CA, USA). This assay, therefore, has the potential to be expanded to incorporate an RT step to make this assay a single tube test. Further testing, however, will be required to validate this assay for use as an OIE/WHO prescribed test, and it is likely that additional advances in technology will be required if the ultimate aim of having a sensitive diagnostic assay which differentiates between viruses is achieved (Fooks et al., 2009). Despite the lack of ability of the SYBR^®^ Green (Applied Biosystems, Foster City, CA, USA) assay to differentiate species, the simplicity of this assay makes this an attractive option for laboratory use as a screening surveillance tool, enabling further analysis by hnRT-PCR or examination of the real-time PCR product by cloning and sequencing.

The current WHO “gold standard” test is the fluorescent antibody test (FAT), which uses a conjugated monoclonal antibody against the RABV nucleoprotein, but is believed to detect all viruses. Developing a sensitive real-time PCR assay capable of detecting all bat and phylogroup II *Lyssaviruses* is particularly important, given that there is a considerable lack of understanding regarding the ecology of most bat *Lyssaviruses* and that of MOKV. Indeed, the reservoir of MOKV is still unknown, despite it being a zoonotic infection leading to human deaths. The assay described should allow the detection of low levels of viral nucleic acid for which current vaccines offer no protection and may be used as a tool for the surveillance of phylogroup I, II and III *Lyssaviruses* in a range of hosts where active infection is suspected. The FAT, whilst very useful and able to detect nucleoprotein, it is not as sensitive as the nucleic-acid detecting hnRT-PCR described by Heaton et al. (1997). Therefore, the development of this assay, approximately 200-fold more sensitive than the hnRT-PCR, may allow better estimation of the true number of cases of MOKV, DUVV and LBV infection. Further studies are required to assess the sensitivity and specificity of this assay, however, this assay already may be useful, where real-time technology exists. For example, the universal primer pair has already been used with species specific probes (Wakeley et al., 2005) for use in an OIE/WHO reference laboratory.

## Figures and Tables

**Table 1:** *Lyssaviruses* used in this study.

Isolate laboratory reference	Isolate GenBank reference	Country of origin	Animal of origin (latin names are given for wildlife, and all are bats except where stated)	Year of isolation	*Lyssavirus* species and reference name
RV1	U22842	Nigeria	*Eidolon helvum*	1956	LBV (b); LBVNig56
RV2	AY062072	South Africa	*Epomophorus wahlbergi*	1980s	LBV (c)
RV3	AY062073	South Africa	*Epomophorus wahlbergi*	1980s	LBV (c)
RV4	AY062074	Nigeria	Shrew (*Crocidura* spp)	1968	MOKV
RV5	AY062077	South Africa	Domestic cat	1970	MOKV
RV20	AY062085	Denmark	*Eptesicus serotinus*	1986	EBLV 1
RV39	AY062075	Cameroon	Shrew (*Crocidura* spp)	1974	MOKV
RV40	AY062076	Central African Republic	*Lophuromys sikapusi* (rodent)	1983	MOKV
RV41	AY339890	Senegal	*Eidolon helvum*	1985	LBV (a), LBVSen85
RV42	EU293117	Cameroon	Shrew (*Crocidura* spp)	1974	MOKV
RV43	EF547449	Central African Republic	*Micropteropus pussilus*	1974	LBV (c), LBVCAR74
RV131	AY062080	Zimbabwe	*Nycteris thebaica*	1986	DUVV, DUVV86
RV133	EF547450	Zimbabwe	Domestic cat	1986	LBV (c), LBVZim86
RV134	EF547455	South Africa	Domestic cat	1982	LBV (c), LBVSA82
RV139	AY062081	South Africa	Bat (possibly *Miniopterus natalensis*^a^)	1981	DUVV
RV175	FJ465418	Zimbabwe	Domestic cat	1981	MOKV
RV611	AY33110	Ethiopia	Domestic dog	1982	LBV (c), LBVEth89
RV628	U89478	UK	*Myotis daubentonii*	1996	EBLV 2
RV634	AF006497	Australia	*Pteropus alecto*	1996	ABLV
RV767	EF547449	France (originally Egypt or Togo)	*Rousettus aegyptiacus*	1999	LBV (a), LBVFra99
RV994	JN016749	South Africa	Domestic dog	2000	RABV
RV1021	FJ465414	South Africa	Domestic cat	1996	MOKV
NA	EF61426	Tajikistan	*Myotis daubentoni*	2001	KHUV
NA	EF614260	Russia	*Murina leucogaster*	2002	IRKV
NA	EF614259	Kyrgyzstan	*Myotis blythi*	1991	ARAV
NA	EF614258	Russia	*Miniopterus schreibersi*	2002	WCBV
NA	GI291195467	Kenya	*Hipposideros commersoni*	2009	SHIBV

a Previously this was described as *Miniopterus schreibersii*, however the genus has been reclassified with the African species now named *M. natalensis.*

**Table 2:** Primers used for the qRT-PCR sensitivity study. Primers for the production of full length nucleocapsid gene amplicons for cloning during this study, and primers used in the real-time PCR assays, are described, where R, purine (A/G); Y, pyrimidine (C/T).

Primer	Sequence	Sense	Position	Reference genome
JW12	5′ATGTAACACCYCTACAATG3′	M	55–73	M13215 (Pasteur virus)
LBV N Nig Rev	5′TTATGAGCTCTCTGAATACAC3′	G	1332–1353	U22842
LBV N Sen Rev	5′TCAAGAGCTCTCCGAGTACAC3′	G	1332–1353	AY339890
LBV N SA Rev	5′CTATGAGCTCTCCGAATACAC3′	G	1332–1353	AY062072
DUVV N Rev	5′GGATGAGGTCACTGAGGTCTATT3′	G	1332–1353	AY062081
MOKV N Rev	5′CAGATACGGCTACCTAGTATT3′	G	1332–1353	AY062074
N165 -146	5′GCAGGGTAYTTRTACTCATA3′	G	165–146	M13215 (Pasteur virus)

**Table 3:** PCR assay sensitivity for a range of *Lyssaviruses* tested during this study. RNA was extracted from infected material for the ‘limit of detection’ studies, serially diluted and used to generate cDNA. The same cDNA sample was used for both the hemi-nested PCR and SYBR^®^ Green PCR with the limit of detection dilutions of initial total RNA given for comparison. Nucleoprotein gene plasmid copies or synthetic Ultramer DNA oligonucleotide copy detection limit and RNA generated by *in vitro* transcription (IVT) copy numbers from these plasmids are also given. Not tested is shown by “–”, not available by “NA”.

Isolate reference	*Lyssavirus* species	Limit of detection by hemi-nested RT-PCR (initial μg/μL total RNA used)	Hemi-nested product sequenced	Limit of detection by SYBR^®^ Green PCR (initial μg/μL total RNA used)	Plasmid or synthetic Ultramer DNA oligonucleotide copies detected by SYBR^®^ Green PCR	IVT derived cDNA copies detected by SYBR^®^ Green PCR
RV1	LBV (b)	10^−2^	Yes	10^−4^	50	195
RV2	LBV (c)	10^−2^	Yes	10^−8^	–	–
RV3	LBV (c)	10^−1^	Yes	10^−5^	5	–
RV4	MOKV	10^−5^	Yes	10^−6^	5	1430
RV5	MOKV	10^−5^	Yes	10^−5^	–	–
RV20	EBLV 1	–	–	10^−6^	–	–
RV39	MOKV	10^−5^	Yes	10^−6^	–	–
RV40	MOKV	10^−5^	Yes	10^−6^	–	–
RV41	LBV (a)	10^−4^	Yes	10^−5^	5	25
RV42	MOKV	10^−5^	Yes	10^−6^	–	–
RV43	LBV (c)	10^−3^	Yes	10^−7^	–	–
RV131	DUVV	10^−1^	Yes	10^−3^	5	190
RV133	LBV (c)	10^−3^	Yes	10^−5^	–	–
RV134	LBV (c)	10^−4^	Yes	10^−5^	–	–
RV139	DUVV	1	Yes	10^−2^	–	–
RV175	MOKV	–	–	10^−7^	–	–
RV611	LBV (c)	1	Yes	10^−2^	–	–
RV628	EBLV 2	–	–	10^−6^	–	–
RV634	ABLV	–	–	10^−6^	–	–
RV767	LBV (a)	10^−3^	Yes	10^−6^	–	–
RV994	RABV	–	–	10^−7^	–	–
RV1021	MOKV	–	–	10^−7^	–	–
NA	KHUV	–	–	–	5	–
NA	IRKV	–	–	–	5	–
NA	ARAV	–	–	–	5	–
NA	WCBV	–	–	–	5	–
NA	SHIBV	–	–	–	5	–
